# Role of radiological corroboration in a locally aggressive inverted papilloma: a case report

**DOI:** 10.1097/MS9.0000000000001193

**Published:** 2023-08-14

**Authors:** Asim Mahat, Gopal K. Yadav, Durga Neupane, Upama Mishra, Sujan Khadka, Bishesh Lamichhane

**Affiliations:** a Department of Radiodiagnosis and Imaging, Nepalese Army Institute of Health Sciences; b Department of Radiodiagnosis and Imaging, Bir Hospital, Kathmandu; c Department of Surgery; d Department of Obstetrics and Gynecology; e Department of Radiodiagnosis and Imaging, B.P. Koirala Institute of Health Sciences, Dharan, Nepal

**Keywords:** benign tumor of paranasal sinuses, case report, inverted papilloma, sinonasal papilloma

## Abstract

**Introduction and importance:**

Sinonasal inverted papilloma is a rare benign tumor of the nasal cavity and paranasal sinuses (PNS). Radiological evaluation is the key to management.

**Case presentation:**

A 46-year-old male presented with complaints of right nasal congestion and occasional bleeding for 4 months. During anterior rhinoscopy, a pinkish fleshy mass occupying the right nasal cavity was seen. X-ray and computed tomography (CT) PNS view showed opacification in the right nasal cavity and maxillary sinus. An MRI of the nose and PNS revealed a peculiar convoluted striated/cerebriform pattern. Histopathology report described the features of an inverted papilloma. The patient underwent endoscopic removal of the mass under general anesthesia. Surgical resection of the tumor was performed along with adjacent normal mucosal tissues. The patient recovered well and was followed-up for recurrence.

**Clinical discussion:**

Sinonasal inverted papilloma is commonly found in males in their fifth to sixth decade of life. A CT scan is the initial modality of choice to evaluate the extent of the disease. MRI is superior to CT in distinguishing tumors from other conditions as well as to evaluate soft tissue extensions. Involvement of the frontal sinus is a risk factor for recurrence. The first option for treating an inverted papilloma is complete surgical removal with the adjacent uninvolved mucosa.

**Conclusion:**

In a biopsy-proven case, radiological assessments like CT and MRI play a pivotal role in studying the typical morphology, delineating the extension, and detecting recurrence.

## Introduction

Highlights

- Sinonasal inverted papilloma is a rare benign tumor of the nasal cavity and paranasal sinuses.
- It has a high recurrence rate and the potential for malignant transformation.
- The multimodal radiological approach is critical in the management of inverted papilloma.

According to the WHO, ʻsinonasal inverted papilloma (SNIP) is a benign epithelial tumor composed of well-differentiated columnar or ciliated respiratory epithelium with variable squamous differentiationʼ of the nasal cavity^1^. Also known as Schneiderian papilloma or Ringertz tumor, it is rare, benign, locally aggressive, and also occurs in the paranasal sinuses (PNS). It has high rates of recurrence and a potential for malignant transformation with chances of converting to malignancy being 5–13%^2^. Among primary nasal tumors, inverted papilloma (IP) accounts for only 0.5–4.0% of the cases^1,3^. SNIP commonly affects males in their fifth to sixth decade^3,4^. Majority of the cases are unilateral, and bilateral involvement has been reported in up to 8% of cases in different series^5^. Other presenting features include epistaxis, rhinorrhea, sinusitis, facial pain or pressure, anosmia, frontal headache, epiphora, proptosis, and otalgia. The duration of signs and symptoms ranges from weeks to decades; the average being 2–3 years. The tumor is friable and often bleeds on manipulation^6^.

SNIP may be difficult to distinguish from other sinonasal mucosal conditions. Histopathology is essential for its definitive diagnosis^5,7,8^. There is a paucity of literature regarding the importance of radiological assessment in SNIP. This study aims to emphasize the value of radiological workups in the case of IP.

In this report, we describe a case of 46-year-old retired military with a history of sinonasal symptoms who was timely diagnosed with benign IP. This study is reported in line with Surgical CAse REport (SCARE) criteria^9^.

## Case presentation

A 46-year-old male presented with complaints of right nasal congestion, rhinorrhea, and occasional nasal bleed for four months. He also had occasional mild to moderate frontal headache and decreased sense of smell. However, his vision was normal and he had no known allergies or prior sino-nasal symptoms. He had no history of facial pain, pressure or numbness, epiphora, diplopia, proptosis, and otalgia. The patient was a nonsmoker and nonalcoholic and had been using an over the counter nasal decongestant which had not relieved the symptoms. Patient denied any past chronic medical illness. During anterior rhinoscopy, a pinkish fleshy mass occupying the right nasal cavity was seen. It was firm in consistency and bled on touch.

**Figure 1:**
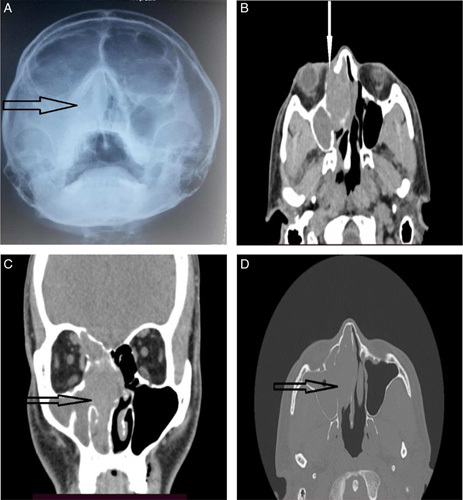
Plain Radiograph of PNS- Water’s view (A), Axial section image of CT PNS soft tissue window (B), Coronal section image of CT PNS soft tissue window (C), Axial section image of CT PNS Bone window (D). PNS, paranasal sinuses.

**Figure 2:**
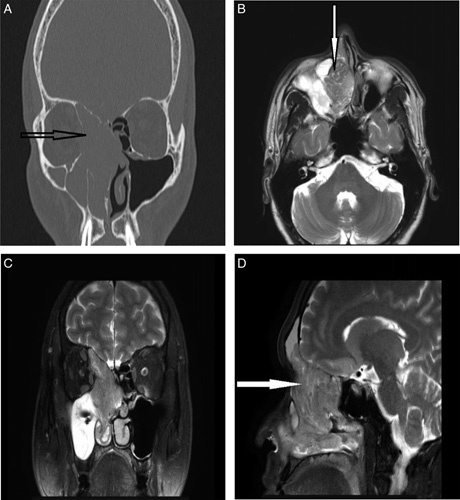
Coronal section image of CT PNS Bone window (A), Axial section image of MRI nose and PNS on T2W sequence (B), Coronal section image of MRI nose and PNS on T2W sequence (C), Sagittal section image of MRI nose and PNS on T2W sequence (D). PNS, paranasal sinuses.

On the same day, he underwent imaging and was followed-up in out-patient basis. An X-ray PNS waters view showed opacification in the right nasal cavity and maxillary sinus (Arrow, Fig. 1A). Noncontrast computed tomography (CT) images acquired from *Toshiba Aquilion* CXL 128-slice CT scanners revealed total soft tissue opacification of the maxillary, ethmoidal complex, and frontal sinus on the right side, merging with the nasal turbinates and obliterating the nasal cavity on the soft tissue window (Figs. 1B and 1C). There was also a widening of the right osteo-meatal complex (OMC) (Arrow, Fig. 1C). The nasal septum deviated mildly towards the left side, and a focal bulge was noted on the lateral aspect protruding into the orbit (Arrow, Fig. 1B). No intracranial extension was noted. On CT PNS Bone window, (Figs. 1D and 2A) thinned out/ rarified nasal septum and erosions of the wall of the involved sinuses were observed. There was remodeling and erosion of the medial wall of the right maxillary antrum (Arrow, Fig. 1D) and lamina papyracea (Arrow, Fig. 2A).

**Figure 3:**
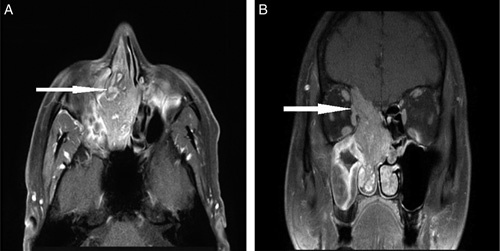
Axial section image of MRI nose and PNS on T1W sequence post enhancement (A), Coronal section image of MRI nose and PNS on T1W sequence post enhancement (B). PNS, paranasal sinuses.

In follow-up after 10 days, MRI images of the nose and PNS acquired from an *Achieva 3.0T Philips* MRI scanner revealed a lobulated, polypoidal mass in the right nasal cavity, with an epicenter in the middle meatus causing widening of the cavity. The lesion appeared heterogeneously hyperintense on T2W images (Figs. 2B, 2C, and 2D) with a convoluted striated/cerebriform pattern (Arrow, Figs. 2B and 2D). Marked heterogenous enhancement with the striated pattern was noted on postcontrast T1W images (Arrow, Fig. 3A). The inferior extension was seen adjacent to the inferior turbinate, which was pushed laterally. The lesion extended into the right maxillary ostium, causing a complete blockage. Retained secretions were also noted within the right maxillary antrum. Superiorly, the lesion extended into the ethmoid and right frontal sinuses causing expansion of the sinuses with retained secretions in the residual right frontal sinus. A protrusion was seen in close approximation with the right medial rectus muscle (Arrow, Fig. 3B).

The patient was admitted on day 12 and planned for endoscopic removal of the mass under general anesthesia. Intraoperative findings corroborated the radiological findings like involvement of the right maxillary, ethmoidal, and frontal sinuses, extension of the papilloma to the floor of the anterior cranial cavity, and involvement of the right medial orbital wall sparing the fat layer. A mucocele was noted in the right maxillary sinus. Surgical removal of the tumor was performed along with adjacent normal mucosal tissues. The mucocele was drained, and an inferior turbinectomy was done. The patient was followed-up for 6 months to look for recurrence. The patient has been recovering well.

The histopathology report revealed that the specimen had hyperplastic stratified squamous epithelium with downward endophytic growth of round to elongated interconnected epithelial nests with a smooth outer contour. These features were consistent with sino-nasal IP without any malignant changes.

## Discussion

Sinonasal papilloma is a histological subtype and originates from the Schneiderian mucosa (an ectodermal derived respiratory epithelium) that lines both the nasal cavity and PNS^4,8^. The pathogenesis of this lesion remains unclear although allergy, chronic sinusitis, and viral infections have been suggested as possible causes^10,11^. Human papilloma virus has been suspected to be a causative agent since the 1980s^12^. The histologic feature distinguishing IP from other mucosal lesions is its propensity to invert and proliferate into the underlying stroma^6^. It typically presents as an obstructing unilateral nasal mass^13^.

Although histopathology is used to confirm the diagnosis, radiology still remains the vital tool for preoperative assessment, to find out the extent, soft tissue involvement and recurrence of the disease. As in the case of sphenoidal sinus IP mentioned by Rabelo *et al*.^14^, where the clinical presentation is often nonspecific and insidious, radiology is the foremost tool for initial diagnosis.

IP cannot be differentiated from other common sino-nasal lesions with an X-ray^2^. As in our case (Fig. 1A), plain radiographs of IP are generally reported as ʻunilateral nonspecific opacification of the maxillary or ethmoid sinus and a mass in the nasal fossaʼ^8^.

Both CT and MRI have a significant role to find out the extention of IP in a biopsy-proven case^3,5^. A CT scan is the modality of choice to evaluate its extent and to visualize bony changes^3,8,13^. On soft tissue window scans, the finding is seen as homogenous opacification of the mass and involved sinuses^8^. This findings are mentioned by Momen *et al*.^13^ in two cases of bilateral SNIPs. Likewise, the right maxillary sinomucosal condition is seen homogenously isodense to the nasal mass in our patient (Figs. 1B and 1C), which is due to poor soft tissue contrast differentiation in unenhanced CT images^7,15^. The constant pressure and mass effect by the papilloma on surrounding bony structures causes bone‑remodeling and erosion, the most common locations being the medial wall of the maxillary sinus and the lamina papyracea^3,5,16^ which is also evident in our findings (Arrow, Figs. 1D and 2A).

Another typical finding caused by obstruction noticed in our case was the widening of the OMC^2,5,16^. In contrast, Head *et al*.^5^ mentioned two cases involving the OMC with contiguous extension into the maxillary sinus. Kader *et al*.^4^ mentioned bony erosion and orbital wall involvement in CT imaging as strong features of malignant IP. However, similar CT findings in our patient were consistent with the benign nature of the papilloma.

MRI is superior to CT in distinguishing tumors from retained secretions, infection, and granulation tissue thus diminishing the chances of overestimating the size of IP^3,8^. In our MR images, we could differentiate the right maxillary sino-mucosal condition from the mass (Figs. 2B and 2C). IP demonstrates a typical pattern on MRI known as a convoluted cerebriform pattern (CCP), which is on T2-weighted or contrast-enhanced T1-weighted MRI is seen as a mix of linear or curvilinear hyperintense and hypointense striations seen in solid components of the tumor^1,3,4,7,8^. It is due to the juxta-imposed epithelial and stromal layers^1^. Our case depicts a similar pathognomonic CCP pattern (Figs. 2B, 2D, and 3A) on T2W and postcontrast T1W MR images, respectively. Various studies have shown that the loss of CCP in IP raises suspicion of malignancy^1,4^. Our case has the involvement of the frontal sinus, which is a rare finding with IP with incidence ranging from 1 to 16% and is considered as a potential risk factor for recurrence^7,15^. MRI is superior to CT while differentiating soft tissue involvement in IP. For instance, we can easily mark the extension of the papilloma in close approximation with the right medial rectus muscle (Arrow, Fig. 3B) which was not possible on CT images.

Complete surgical removal with the adjacent uninvolved mucosa is the first option for the treatment of IP, which minimizes the risk of recurrence^7,8,13,16^. Our case was managed with a similar standard approach. There was a 9% overall rate of malignant transformation from Schneiderian papilloma in the meta-analysis study and therefore, the potential for malignancy of IP should be kept in mind^17^.

## Conclusion

SNIP is a rare, locally aggressive benign tumor with the potential for malignancy and a high recurrence rate. When biopsies confirm the presence of IP, radiological examinations such as CT and MRI are critical for studying the typical morphology, delineating the extension, and detecting any signs of recurrence. The scarcity of radiological literature on SNIP arising from the frontal, ethmoidal, and sphenoid sinuses, as well as imaging findings to distinguish benign from malignant SNIP, requires careful consideration. The primary treatment principle is complete surgical resection with adjacent normal mucosa. Despite the IP’s aggressive nature and destructive characteristics, postoperative recovery is usually excellent. As a case report, our radiological findings may lack generalizability, but they highlight the pressing need for many more such studies in the literature.

## Ethical approval

This is a case report, and the IRB of the Nepalese Army Institute of Health Sciences, which is part of the Nepal Health Research Council, does not require ethical approval for such research, but they do require patient consent.

## Consent

Written informed consent was obtained from the patient for the publication of this case report and accompanying images. A copy of the written consent is available for review by the Editor-in-Chief of this journal on request.

## Sources of funding

None.

## Author contribution

All the authors contributed equally to writing and preparing the manuscript. The final version of the article is approved by all authors.

## Conflicts of interest disclosure

The authors declare that they have no conflicts of interest.

## Research registration unique identifying number (UIN)

Registry used: Research Registry.

Hyperlink : researchregistry9060.

## Guarantor

Asim Mahat.

## Provenance and peer review

Not commissioned, externally peer-reviewed.

## Data availability statement

All data about the case are available as a part of the article and no additional source data are required.
